# UV-Assisted Hyperbranched Poly(β-amino ester) Modification of a Silica Membrane for Two-Step Microfluidic DNA Extraction from Blood

**DOI:** 10.1021/acsami.3c03523

**Published:** 2023-06-15

**Authors:** Akshaya Jagannath, Yinghao Li, Hengji Cong, Jaythoon Hassan, Gabriel Gonzalez, Wenxin Wang, Nan Zhang, Michael D. Gilchrist

**Affiliations:** † School of Mechanical and Materials Engineering, University College Dublin, Belfield, Dublin 4, Ireland; ‡ The Charles Institute of Dermatology, School of Medicine, University College Dublin, Belfield, Dublin 4, Ireland; § National Virus Reference Laboratory, University College Dublin, Belfield, Dublin 4, Ireland; ∥ International Collaboration Unit, Research Center for Zoonosis Control, Hokkaido University, N20 W10, Kita-ku, Sapporo 001-0020, Japan; ⊥ MiNAN Technologies Ltd., NovaUCD, Belfield, Dublin 4, Ireland

**Keywords:** microfluidic, nucleic acid extraction, solid-phase extraction, surface modification, polymer synthesis, poly amine esters, point-of-care testing, integrated systems

## Abstract

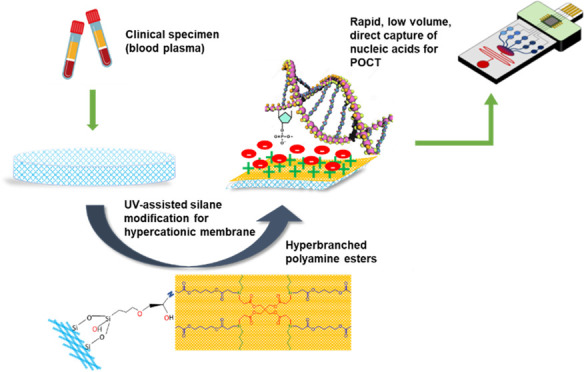

Integrating nucleic acid extraction in amplification-based point-of-care diagnostics will be a significant feature for next-generation point-of-care virus detection devices. However, extracting DNA efficiently on a microfluidic chip poses many technological and commercialization challenges, including manual steps, multiple instruments, pretreatment processes, and the use of organic solvents (ethanol, IPA) that inhibit detection, which is not viable with routine testing such as viral load monitoring of transplant patients for post-operative care. This paper presents a microfluidic system capable of two-step DNA extraction from blood using a UV-assisted hyperbranched poly(β-amino ester) (HPAE)-modified silica membrane for cytomegalovirus (CMV) detection in a rapid and instrument-free manner without the presence of amplification inhibitors. HPAEs of varying branch ratios were synthesized, screened, and coated on a silica membrane and bonded between two layers of poly(methyl methacrylate) (PMMA) substrates. Our system could selectively extract DNA from blood with an efficiency of 94% and a lower limit viral load of 300 IU/mL in 20 min. The extracted DNA was used as the template for real-time loop-mediated isothermal amplification (LAMP)-based detection of CMV and was found to produce a fluorescent signal intensity that was comparable with commercially extracted templates. This system can be integrated easily with a nucleic acid amplification system and used for routine rapid testing of viral load in patient blood samples.

## 1 Introduction

Preemptive antiviral therapy is an important strategy for preventing human cytomegalovirus (HCMV) infection in patients undergoing solid organ transplants. Early-stage detection of HCMV is critical for determining the appropriate treatment for post-operative care.^1^ Nucleic acid amplification tests (NAATs) are a sensitive and specific method for quantifying viral load in a near-patient setting in clinics or laboratories that perform routine testing. One key component of the NAAT process is nucleic acid extraction, which is typically performed using silica or surface-functionalized magnetic beads. However, commercialization of point-of-care systems is hindered by the production and standardization of multiple independent components required for pathogen detection from clinical specimens.^2−4^

Silica is the matrix generally used for solid-phase extraction (SPE) due to its historical use of purifying DNA from agarose gels. The process involves using a lysis buffer containing a detergent to dissolve membrane lipids and proteins, which causes the release of cell contents through pores. The DNA is then exposed to high salt and low pH conditions, leading to the disruption of noncovalent bonds in the phosphate backbone. The surplus of negative charges on the DNA allows it to bind to the positively charged surface of silica through hydrophobic interactions. However, proteins in the lysed cell can nonspecifically bind to these surfaces and can be removed through selective washing with cleaving enzymes, while ethanol can maintain the bound DNA to the surface. When the pH is increased, the negative charge density on the membrane surface results in greater electrostatic repulsion between the DNA and the silica surface, causing elution.^5−7^ The wide adaptation of silica surfaces is due to its stability, biocompatibility, and easy modification properties in solid-phase extraction. However, a common problem is the carry-over of residual chaotropic salts and ethanol into the amplification reaction, which can inhibit polymerase amplification.^8^

Although silica spin column methods are used in laboratory settings, which have more resources and equipment, they present a myriad of challenges in point-of-care technology integration. These methods are labor-intensive (>20 steps, multiple reagents, multiple wash/centrifuging cycles), time-consuming (1–2 h on average), and restricted to certain specimens or types of nucleic acids (separate products for extraction from saliva, blood, etc.). This greatly limits their potential in one-pot systems. To overcome these limitations, microfluidic-based point-of-care devices have been developed that can accommodate on-chip SPE using versatile silica structures: silicon-based micropillars,^9−11^ silicon nanowires,^12^ rotating microfluidic systems using silicon beads,^13^ and monodisperse-porous silica microspheres^14^ on the microfluidic chip.

Recent advances in on-chip nucleic acid extraction from blood include enhanced solid-phase extraction by combining ultrasonic cell lysis with silica membrane-based SPE for rapid sample preparation from serum samples.^15^ Another method involves surface modification of the plastic substrate by introducing additional positive charges to facilitate DNA capture via electrostatic binding or complexing with the cationic polymer.^16,17^ For example, poly(2-dimethylaminomethyl styrene) films can be deposited on poly(ethylene terephthalate) (PET) via an initiated chemical vapor deposition process to introduce positive charges on the inner surfaces of the microchip. For these systems, the microchip requires ∼30 min of incubation to capture 90% DNA from cell lysate. Alternatively, another method using polymer monolith columns can be impregnated with silica particles to form a silica–polymer composite to directly extract nucleic acids from blood.^18^ Here, the silica-impregnated porous polymer monolith columns could also generate mechanical shear to assist the buffer-based lysis of cells. Increasing the number of positive charges through innovative sample extraction systems on the chip, such as a dimethyl adipimidate/thin-film system^19,20^ or a homobifunctional imidoester (HI)-modified plastic surface,^21^ has resulted in the rapid and efficient capture of nucleic acids from blood. However, the nonspecific binding to any negatively charged molecules present in the sample is a major drawback of these systems. Additionally, the detection of trace amounts of pathogens in clinical samples is challenging. For early detection, processing of whole blood or plasma in a microfluidic device without pathogen enrichment may not yield an amplifiable signal.^22−26^ Commercially adaptable sample extraction systems require the ease of automation integration with amplification and detection, minimizing manual steps, and the use of external instruments. Currently, these demands are hindered by high sample volume requirements (>1 mL), manual steps such as cartridge transfer, additional instrumentation, and increased duration (2–3 h).^27−30^ Thus, there is a need for a specific and efficient cationic polymer system that can integrate with microfluidic platforms for rapid nucleic acid capture from clinical samples.

Poly amine esters (PAEs) are widely used in gene therapy and drug delivery.^31^ Compared to the conventional synthetic polymers used in DNA capture that always require a strict or complex polymerization process, PAEs are easier to synthesize.^32^ Synthesis can be achieved by facile Michael addition, making it more suitable for large-scale manufacture. Meanwhile, the abundance of inexpensive monomers and easily modified terminal groups allows for tuning of the polymer chemical structure and topology according to application requirements. In addition, the DNA purity and concentration are measured by calculating the absorption ratio at 260 nm (conjugated purine and pyrimidine groups) and 280 nm (conjugated amino acids). As the PAE backbone does not have a conjugated structure, it does not interfere with absorption measurements at shorter wavelengths; thus, the residual coating polymer in the DNA extract did not affect the absorption values. To construct PAEs with high DNA capture capability, the monomer combination of 1,4-butanediol diacrylate (B4), 5-amino-1-pentanol (S5), and 3-morpholinopropylamine (MPA) was selected. According to previous research, the combination of hydrophobic B4 and hydrophilic S5 can facilitate strong DNA binding capacity while maintaining stability in the aqueous environment.^33^ Meanwhile, MPA is a group proven to have strong DNA interaction ability. The terminal modification of MPA can enhance the DNA binding capability of the PAEs.^34^ Moreover, a branch unit, pentaerythritol tetraacrylate (PTTA), was introduced to achieve a hyperbranched PAE, resulting in a more pending terminal.^35^ The transition from a linear to a branched structure enhances the DNA binding ability of PAEs, which is particularly important given the difficulty in achieving high-molecular-weight linear PAEs with a limited number of terminal groups.

**Figure 1:**
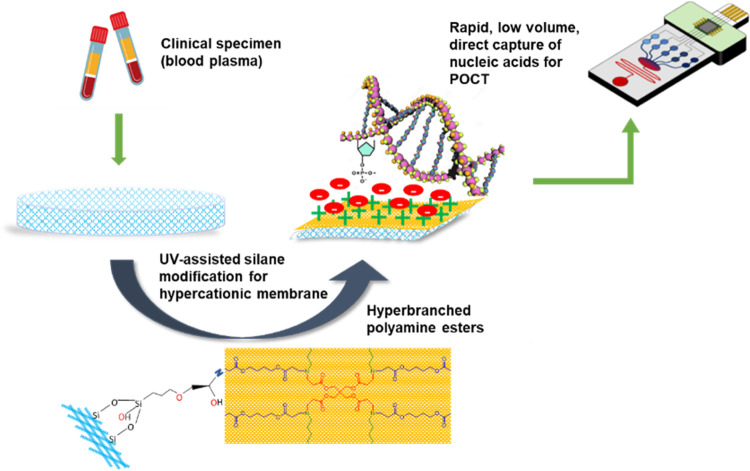
Direct capture of DNA from a clinical specimen on an HPAE-modified Si membrane for rapid, low-volume detection on point-of-care devices.

In this paper, we present a novel application of hyperbranched poly(β-amino ester)s (HPAEs) in modifying the surface of a silica membrane to increase the electrostatic capture of DNA from low-volume blood plasma on a microfluidic device. This application adds to the aforementioned advantages of the silica membrane and facilitates extraction without the need for organic solvents or additional instruments (Figure 1). HPAE is attached to silica fibers in the membrane using an organosilane cross-linking agent, which can then be easily embedded in between two layers of poly(methyl methacrylate) (PMMA) chips for extraction. The resulting extraction chip can process the sample in an instrument-free, direct (two steps, no lysis/pretreatment), and rapid (<20 min) manner to integrate with an amplification component for pathogen detection. By bonding multiple layers of PMMA with the embedded HPAE-modified membrane, the plasma sample can be incubated with the membrane for selective DNA binding on the top layer, filtered through to the bottom layer, and eluted out by pressure-driven flow. The assay optimization of this membrane-based device does not contain organic solvents such as ethanol, thus removing the possibility of amplification inhibitors. The highly cationic nature of the modified membrane is significant for the direct binding and elution of DNA without the use of multiple components. The eluted DNA is amplified by using real-time loop-mediated isothermal amplification (LAMP), and the fluorescent signals are recorded. LAMP-based detection was selected to amplify the extracted DNA due to the highly specific nature of the assay (six primers or regions of complement), isothermal requirements (rapid and no thermocycler needed), and the ease of signal detection and viral load quantification through fluorescently labeled loop primers.^36,37^

## 2 Materials and Methods

### 2.1 Polymer Synthesis for Membrane Coating

#### PAE Material Synthesis

Chemicals 1,4-butanediol diacrylate (B4), 5-amino-1-pentanol (S5), 3-morpholinopropylamine (MPA), pentaerythritol tetraacrylate (PTTA), lithium bromide (LiBr), deuterated chloroform (CDCl_3_), and dimethyl sulfoxide (DMSO) were purchased from Sigma-Aldrich (U.K.). Solvent dimethylformamide (DMF) and diethyl ether were purchased from Fisher. Poly(β-amino ester)s (PAEs) were synthesized using different amounts of monomers (Table 1) dissolved in DMSO and the reaction was performed at 90 °C. Gel permeation chromatography (GPC) was used to monitor the growth of molecular weight. Once the weight molecular weight (*M*_w_) reached the target value, the reaction was further diluted with DMSO and end-capped with MPA. Finally, the polymer product was precipitated in diethyl ether and dried under vacuum to remove solvent residue.

**Table 1:** Monomer Amount for the Synthesis of PAEs

	B4 (g)	PTTA (g)	S5 (g)	MPA (g)
E12	0.99	0.18	0.48	1.12
E13	0.99	0.18	0.49	1.08
E14	0.99	0.18	0.50	0.95
E15	0.99	0.18	0.52	0.86
E17	1.19	0	0.52	0.86
E18	1.19	0	0.56	0.84
E19	1.19	0	0.60	0.84

#### Molecular Weight Measurements

The number average molecular weight (*M_n_*), weight average molecular weight (*M*_w_), and polydispersity index (PDI) of polymers were determined by GPC equipped with a refractive index detector (RI), a viscometer detector (VS DP), and a dual-angle light scattering detector (LS 15° and LS 90°). To monitor the *M*_w_ of polymers during the polymerization process, 20 μL of the reaction mixture was collected at different time points, diluted with 1 mL of DMF, filtered through a 0.2 μm filter, and then measured by GPC. The two in-series columns were eluted with DMF and 0.1% LiBr at a flow rate of 1 mL/min at 60 °C.

#### Proton Nuclear Magnetic Resonance (^1^H NMR)

The chemical structure and composition of polymers were confirmed with ^1^H NMR. PAEs were dissolved in CDCl_3_. Measurements were carried out on a Varian Inova 400 MHz spectrometer and reported in parts per million (ppm).

### 2.2 Membrane Preparation

**Figure 2:**
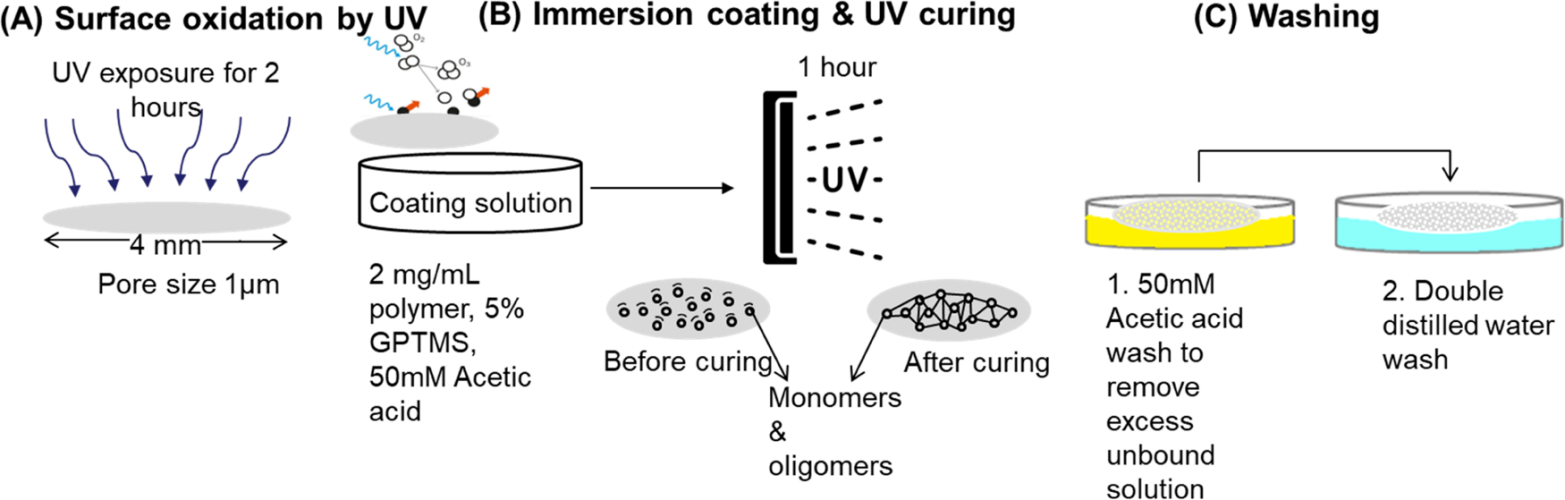
PAE polymer-coated membrane preparation process: (A) silica membrane is cut, sterilized by ethanol wash, and dried before exposure to UV for surface oxidation for 2 h. (B) UV-treated membrane is immersed in a coating solution containing 5% GPTMS, 2 mg/mL synthesized PAE, and 0.05 M acetic acid^38,39^ and cured under UV for 1 h (C) The modified membrane is washed in a 50 mM acetic acid solution and double-distilled water to remove the excess unbound polymer and dried overnight.

Commercial silica membrane sheets for nucleic acid extraction with a pore size of 1 μm and a thickness of 1 mm were purchased from Biocomma Ltd. (Shenzhen, China). The membranes were cut, washed, and dried with 70% ethanol (Fisher Scientific). To modify the surface, (3-glycidyloxypropyl)trimethoxysilane (GPTMS) and acetic acid were purchased from Sigma-Aldrich (U.K.). All experiments were carried out in a Biobase BSL-II laminar air flow chamber. The surface of the commercial Si membrane was oxidized by UV exposure, followed by direct adsorption of the polymer solution containing the HPAE polymer and the cross-linker in varying amounts.^38,39^ After curing the membrane under UV, the excess PAE solution was washed out and dried overnight in a desiccant chamber (Figure 2). To identify optimum conditions under which the surface modification of the polymers on the membrane was durable and able to extract DNA, surface oxidation time, GPTMS amount in the polymer solution, and polymer concentration were all varied (Table 2). Characterization of the modified membrane was performed using a Bruker ATR-FTIR system and imaged on a Zeiss Sigma 300 SEM.

**Table 2:** Coating Conditions for the PAE-Modified Si Membrane for Extraction

parameters	values
UV exposure	15/30 min/1/2 h
GPTMS concentration	0/1/5/10%
polymer concentration	1/2/10/20/40 mg/mL

### Microfluidic Chip Fabrication and Assembly

The chips were laser cut from industrial-grade PMMA sheets (Radionics Ltd.) and milled to a tolerance of ±100 μm in the microchannels using a Microcut 873 milling machine. Analytical-grade isopropanol and chloroform were purchased from Sigma-Aldrich for the ultrasonication bath and thermal bonding, respectively. Polymer–polymer bonding of the cleaned chips was performed in a precision hot press (MiNAN Technologies). Milled PMMA chips were first cleaned in an isopropanol bath under ultrasonication for 3 min and dried using an air gun. The bonding sides were exposed to UV/ozone treatment for 5 min, followed by direct chloroform deposition for 2 min.^40^ Immediately afterward, the bonded layers were subjected to high-pressure bonding at 6000 N at 63 °C for 10 min.

### Extraction Assay and Fluorescent LAMP-Based Detection

Washing and elution buffer ingredients (guanidinium isothiocyanate, Tris-HCl, Triton X, EDTA) of molecular grade were purchased from Sigma-Aldrich (U.K.). Blood plasma was obtained from the National Virus Reference Laboratory, Dublin, Ireland, and the plasma was spiked to the required viral load using Acrometrix CMV Standards (300 to 300,000 IU/mL). Total genomic DNA was extracted using a Qiagen (Thermo Fisher) QiaAMP Virus MiniElute Spin Kit. Absorption at 260 and 280 nm for assessing DNA purity and yield were measured using a Thermo Scientific NanoDrop Lite Spectrophotometer. DNA purity % and DNA recovery % were estimated five times for each condition and recorded with the standard deviation according to the equations below.
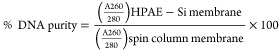

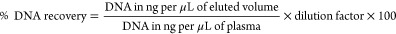
For LAMP-based detection, WarmStart fluorescent LAMP master mix and primers were purchased from New England Biolabs, U.K., and the primers were designed using the NEB LAMP Primer Design Toolkit with specific modifications to target the viral DNA polymerase region UL54, which was inhibited by the activated first-line therapeutic drug, ganciclovir, and thus frequently used in CMV infection treatment.^41^ The loop forward primer was fluorescein tagged at the 3′ end for signal detection. Nuclease-free water (molecular-assay grade) purchased from Thermo Fisher was used for all assays. An Applied Biosystems QuantStudio 7 Flex Real-Time PCR System was used to detect the FAM-6 signal. The amplification program was run for 45 min at 65 °C, followed by heating at 85 °C for 5 min. Two sets of standard positive controls (Acrometrix CMV 3000 IU/mL) extracted using Qiagen MiniElute and no template control (nuclease-free water) were run during each assay, and the amplification signals were recorded on QuantStudio QS7 Real-Time PCR Software. The median viral load in transplant patients with CMV infection was found to be ≥1000 copies/mL or 582 IU/mL.^42,43^ Thus, for determining the lower limits of detection, the clinically relevant range of viral loads was considered for testing from 300 IU/mL to 300,000 IU/mL using standard Acrometrix CMV controls.^44^ All buffers were adjusted for their appropriate pH, using a digital pH meter.^8,45−47^ Assay optimization for the on-chip process was performed to identify factors of significance, while the membrane size was fixed (Table 3).

**Table 3:** On-Chip CMV DNA Extraction from Spiked Blood Plasma: (A) Chemical Composition of Washing and Elution Buffers; (B) Assay Optimization Factors; and (C) LAMP Primer Sequences for Detection of the UL54 Region in Human CMV

A	washing buffer		elution buffer	
	GuSCN	4 M	Tris-HCl	10 mM
	Triton X	20 g/L	EDTA	0.1 mM
	Tris-HCl	10 mM		
		pH adjusted to 6.5 (1 N HCl)		pH adjusted to 8.5 (1 M NaOH)

B	parameters	values
	viral load	3000 IU/mL in blood plasma
	input blood volume	20/50/100/200 μL
	sample-to-buffer ratio	1:1/1:2 /1:4
	incubation	20/56 °C
	elution buffer volume	50/100/200 μL
	flow rate	20/40/80/160 μL/min

C	sequence name	sequence
	CMV_UL54S_F3	GACCATGGCCAGAAAAATCGGCGATCCACACGGACCTCGT
	CMV_UL54S_B3	ACACTGCAAATCGGGCACGGCATACGCCGTGTCTGTCAC
	CMV_UL54S_LF	GCAGGGTTTTCCCG(**Fluorescein-dT**)G
	CMV_UL54S_FIP	GACCATGGCCAGAAAAATCGGCGATCCACACGGACCTCGT
	CMV_UL54S_BIP	ACACTGCAAATCGGGCACGGCATACGCCGTGTCTGTCAC
	CMV_UL54S_LB	GCGTCCCGTACCCGTAGAT

## 3 Results and Discussion

### 3.1 Polymer Synthesis and Characteristics

**Figure 3:**
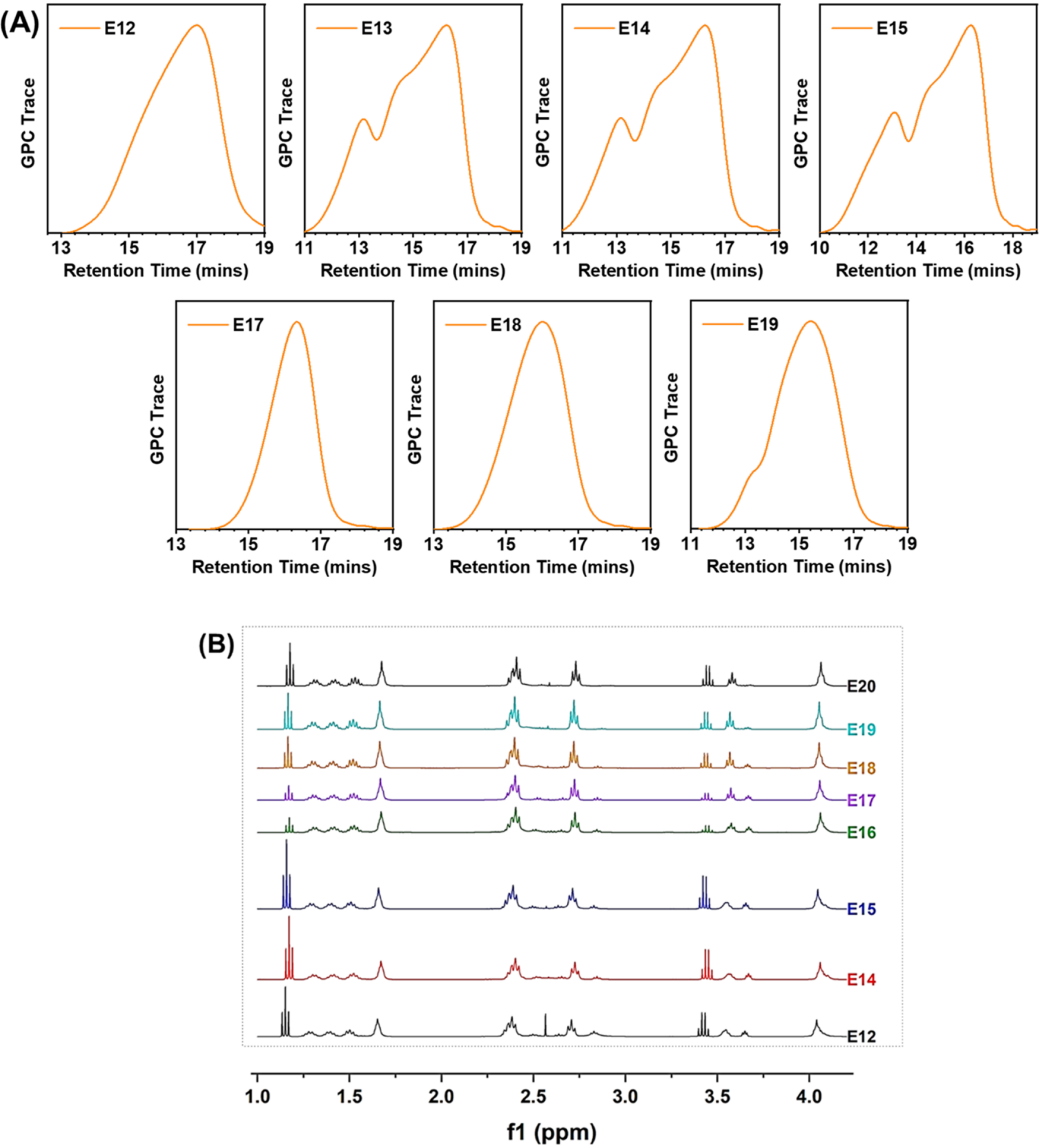
Characterization of synthesized PAEs: (A) GPC traces and (B) ^1^H NMR spectra.

**Scheme 1:**
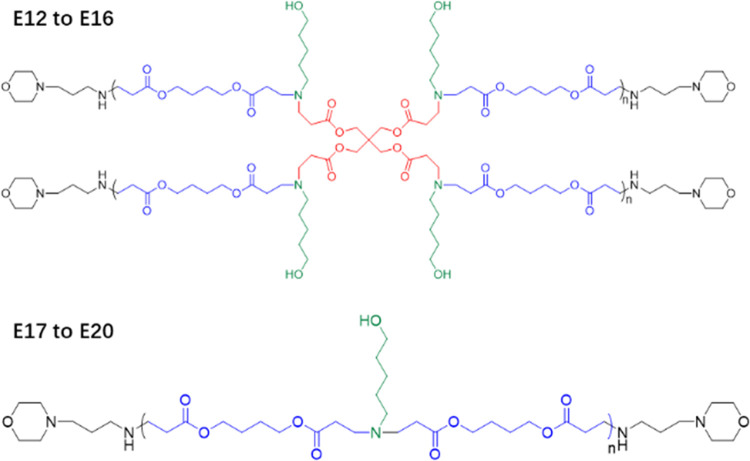
Chemical Structure of PAEs

To tailor the properties of the poly(β-amino ester)s (PAEs) for our application, a series of HPAEs, PAEs E12 to E19 with the branch ratio (branch monomer to the linear monomer molar ratio) as 0.1, were designed (Scheme 1). According to the GPC results (Figure 3 and Table 4), their molecular weights (*M*_w_) range from 10.7 to 64.0 kDa. Meanwhile, a group of linear PAEs, E17 to E20 with molecular weights (*M*_w_) ranging from 5.3 to 21.1kDa, were synthesized as a comparison. The polymer structures and monomer combinations were confirmed by NMR (Figure 3B and Table 4A). Our NMR analysis shows that the number of terminal amines (MPA) increases in proportion to *M*_w_ in the branched PAEs (E12 to E15), while the opposite trend is observed in the linear PAEs (E17–E20). Since the terminal amines have abundant nitrogen and oxygen groups, they have a higher chance of interacting with the DNA and silica membrane. Additionally, the PAEs with higher molecular weights are more hydrophobic, which means they are more stable in water than the low-molecular-weight products. Based on these factors, the PAEs (E15) with the highest molecular weight and the greatest number of terminal groups are hypothesized to exhibit the best performance.

### 3.2 HPAE-Modified Si Membrane Characteristics

**Figure 4:**
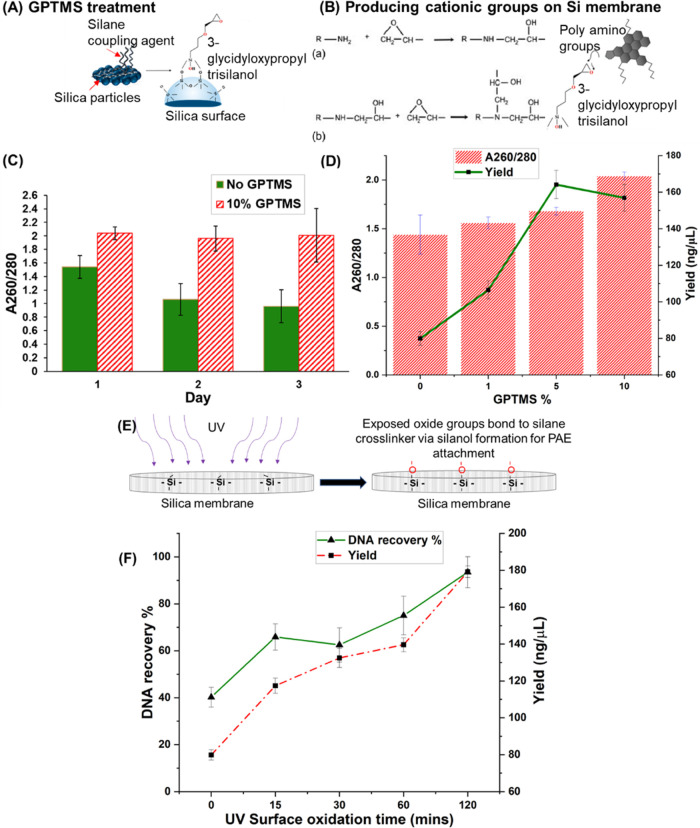
Effect of surface oxidation and silanization through UV and GPTMS treatment on DNA yield and purity. (A) GPTMS treatment of the oxidized Si surface forming silanol bonds. (B) Primary amines (a) and secondary amines (b) react with epoxy groups of GPTMS. (C) Absorbance ratio of DNA to contaminants after extraction using membranes with or without 10% GPTMS retained over time (*p* < 0.05). (D) Increasing GPTMS % in the membrane improves DNA capture as the absorbance ratio improves during extraction and higher resultant yield (*p* < 0.05). (E) Exposed oxides on Si groups on the membrane surface. (F) Increase in time for oxidation increases purified DNA yield and absorbance due to increased cationic groups on the surface (*p* < 0.05).

The epoxy groups on the GPTMS form a covalent bond with the primary and secondary amines of the HPAE polymers (Figure 4A,B) and bind to an oxidized silica surface.^39,48,49^ It was found that the volume of the cross-linker GPTMS in the HPAE polymer solution is significant for the shelf life of the modified membrane. Without any GPTMS, the recovery and purity of DNA reduce by 50%, while DNA recovery increases with an increase in the percentage of GPTMS (10% by volume) in the polymer coating solution (Figure 4C). The increase in GPTMS volume leads to a higher number of cationic groups on the Si membrane, providing more binding sites for the negatively charged phosphate backbone of DNA (Figure 4D). However, using more than 10% GPTMS in the coating solution was found to inhibit downstream amplification processes, resulting in the signal intensity of CMV LAMP below the threshold for DNA detection. Despite the relatively high yield of DNA (Figure 4D), a cloudy precipitate was observed in the extracted DNA at 10% GPTMS, which could be the excess silanes. To avoid this issue and not affect LAMP detection, further experiments were carried out using 5% GPTMS.

Oxidation of the silica membrane can be carried out by chemicals such as treating the surface with piranha solution or physical methods like UV exposure. Exposure of silica fibers to UV for a prolonged time increases the number of oxidized Si groups on the surface (Figure 4E). This, in turn, promotes HPAE polymer binding on adsorption with the cross-linker. A linear increase in DNA absorbance and yield is observed with the surface oxidation time of membranes before HPAE coating. With a higher exposure time to UV, the number of oxidized silica sites on the membrane increased, enabling a greater number of GPTMS-linked HPAE molecules to attach to the surface through their epoxy groups. This is further supported by the increase in DNA yield from 117.43 to 179.18 ng/μL, as shown in Figure 4F.

**Figure 5:**
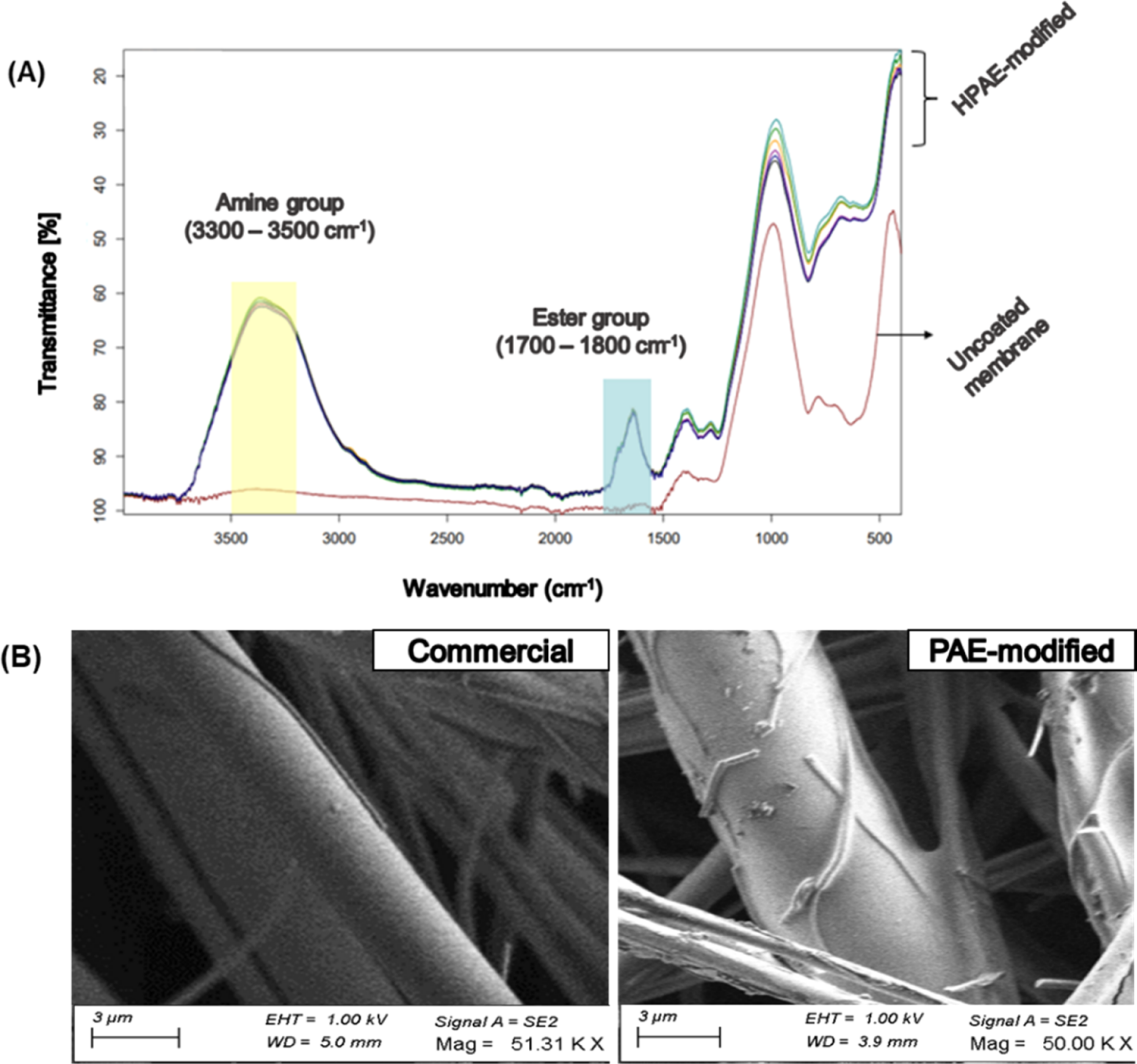
Analysis of commercial (uncoated Si membrane) and HPAE-modified (modified Si membrane) membranes confirming the presence of (A) amine and ester groups in the Si membrane modified by E12–E20 HPAEs. (B) Polymer structures on the modified surface of membrane fibers observed and compared against smooth membrane fibers (commercial).

Attenuated total reflection-Fourier transform infrared (ATR-FTIR) spectra confirmed the presence of amine and ester groups on membranes modified using all of the polymers (E12–E20) using 5% GPTMS, 2 h UV exposure, and 5 mg/mL HPAE polymer concentration. The FTIR analysis was repeated for 5 days consecutively to observe any reduction in transmittance peaks at 3300–3500 and 1700–1800 cm^–1^ and was observed to be consistent. As the number of amine groups was significantly higher than that of the esters, the transmittance peak at wavelengths corresponding to primary and secondary amine groups was also much higher (Figure 5A). SEM analysis of membrane fibers before and after modification revealed fold-like structures on the surface of the fibers, which were smooth before modification. These structures are likely the HPAE particles attached to the surface after modification (Figure 5B). To ensure there was no dust or contamination on the surface, the membranes were modified in a sterilized laminar flow chamber and placed carefully between two clean glass slides before imaging. The imaging was repeated three times in two different SEM instruments to avoid any artifacts. Imaging of the same samples was done after one month to observe if the HPAE structures remained on the surface; they were found to remain consistent.

**Table 4:** (A) Structure Properties of PAEs and (B) Monomer Molar Ratios of HPAEs

A		*M_n_*	*M*_w_ (Da)	PDI	branch ratio
	E12	4475	10,707	2.4	0.1
	E13	6447	38,170	5.9	0.1
	E14	6289	42,246	6.7	0.1
	E15	6641	63,991	9.6	0.1
	E17	3555	5294	1.5	0
	E18	4513	7996	1.8	0
	E19	6938	21,125	3.0	0

B		B4 (g)	PTTA (g)	S5 (g)	MPA (g)
	E12	1	0.1	0.94	0.28
	E13	1	0.1	0.96	0.32
	E14	1	0.1	0.98	0.38
	E15	1	0.1	1	0.40
	E17	1	0	0.83	0.34
	E18	1	0	0.91	0.18
	E19	1	0	0.97	0.06

### 3.3 Microfluidic Assembly and On-Chip HPAE-Modified Membrane Extraction

**Figure 6:**
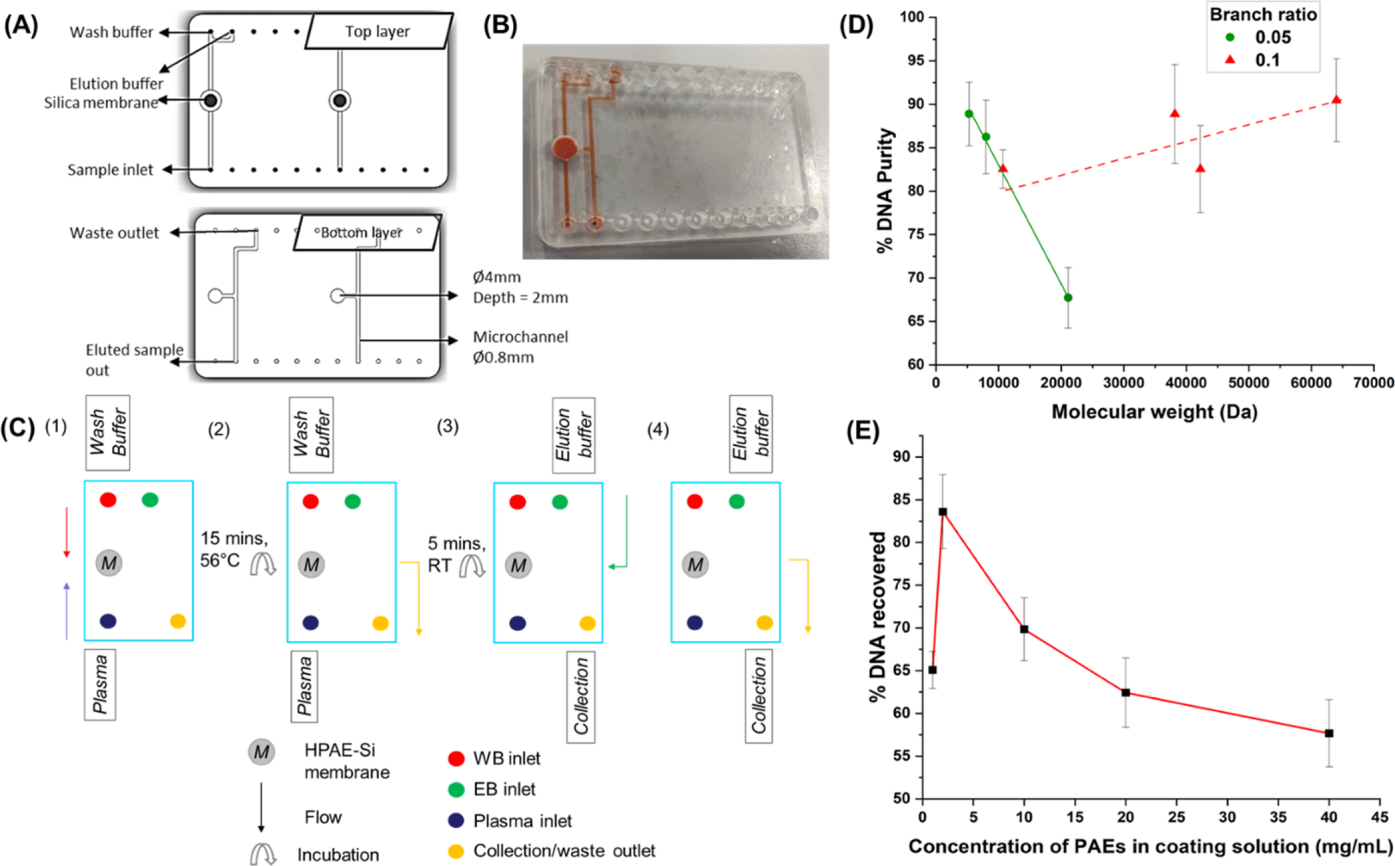
Design and testing of the bonded microfluidic extraction chip with the PAE-modified membrane: (A) Sample is pumped through the inlet port into the embedded PAE–silica membrane and filtered into the bottom layer. (B) Dye injection to test against leakage/blocks in microchannels. (C) DNA extraction assay: 50 μL of the sample and 100 μL of wash buffer are injected into the modified membrane via ports and incubated for 15 min at 56 °C before elution. (D) Increasing cationic binding sites in higher PAE molecular weight results in increased yield for branched polymers (*p* < 0.05)**. (E) Effect of PAE concentration in membrane modification solution on the quality of extractant (*p* < 0.05)**.

The bonded microfluidic chip containing the membrane was designed (Figure 6A) and analyzed for fabrication defects using a standard microscope. To ensure there was no leakage or structural disturbances to the fluid movement within the sealed chips, a locally purchased food dye was injected into the ports and the flow was tracked using a microscope (Figure 6B). All of the channels were properly filled, and the test was repeated over four separate bonded chips for confirmation. The bonding strength was assessed using burst pressure test equipment. A manual water pressure test pump was used to pump water into the inlet of the chip while blocking the outlet with a plug. This plug was manually pressed during the test, and the pressure gauge was used to show the burst pressure of the chip. Four different chip sets were assessed, and bonded chips did not produce leakage at a maximum pressure of 50 MPa.

On-chip extraction was carried out using blood samples spiked with Acrometrix CMV standards (3000 IU/mL). 50 μL of CMV spiked plasma samples were injected through the sample inlet port using a sterile syringe and a connecting tube. After incubation on a thermal heating block, 100 μL of elution buffer was pumped in and the purified DNA was collected and analyzed using a Nanodrop Spectrophotometer (Figure 6C).

To achieve good DNA extraction, the coating PAEs with different structures were screened. The evaluation of DNA purity on PAE-coated membranes revealed an essential relationship between coating PAE’s molecular weight and the molecular structure with DNA extraction efficiency. Figure 6D illustrates that in linear PAEs E17 to E19, the DNA purity decreased rapidly with the increase of molecular weight (as the molecular weight increased from 5k to 21k, the DNA extraction efficiency decreased from 88 to 67%), which was due to the reduction of the terminal amine group in high-molecular-weight PAEs. This molecular weight-dependent tendency indicated that the terminal functional groups of the polymer could significantly help DNA extraction. Specifically, the pending positively charged terminal amines can interact and bind DNA via electrostatic interaction. Since HPAE has more amino terminations than LPAE, different HPAE-coated membranes were investigated further. As expected, HPAE can significantly improve the DNA extraction efficiency of membranes—all of the tested groups maintained more than 80% DNA purity. In addition, with the increase of coated-HPAE molecular weight, the membranes’ DNA extraction performance improved slightly (82 to 90%, *M*_w_ from 10k to 64k), which is consistent with the previous prediction. However, the limited improvement in efficiency from E12 to E15 shows that the positively charged termination density in the coating polymer is the main factor affecting DNA extraction performance. In comparison with LPAE, the positive charge density in HPAE does not change with the increase of molecular weight. Based on the above results, HPAE E15 exhibited the best performance in the coating, making it highly advantageous for future device production. Specifically, HPAE is not sensitive to molecular weight, allowing for consistent performance across a wide range and eliminating the need for precision instruments such as GPC to monitor large-scale production. In addition, HPAE has a shorter synthesis time than linear PAE. It is optimized to complete the HPAE polymerization reaction in hours, much faster than the days required for LPAE. Most importantly, compared with other publications,^50^ the synthesis conditions of HPAE are mild (do not require high temperature/low temperature, high pressure/vacuum, or even protection of inert conditions). Consequently, no large, specialized equipment is required to produce the coatings, making them easy to manufacture. Moreover, the reactant can have a reaction extent close to 100%, thus guaranteeing a very high yield. All these merits show that the HPAE-coated membrane can work as a promising DNA extraction device.

To optimize the coating conditions, E15 solutions with different concentrations were tested (Figure 6E), and it was found that the PAE-based membrane exhibited the best performance after treatment with a 2 mg/mL solution. However, higher concentrations of PAE led to a decrease in DNA recovery, likely due to a higher number of attached polymer molecules, resulting in less DNA release during the assessment.

### 3.4 On-Chip Assay Optimization and FLOS-LAMP Detection

**Figure 7:**
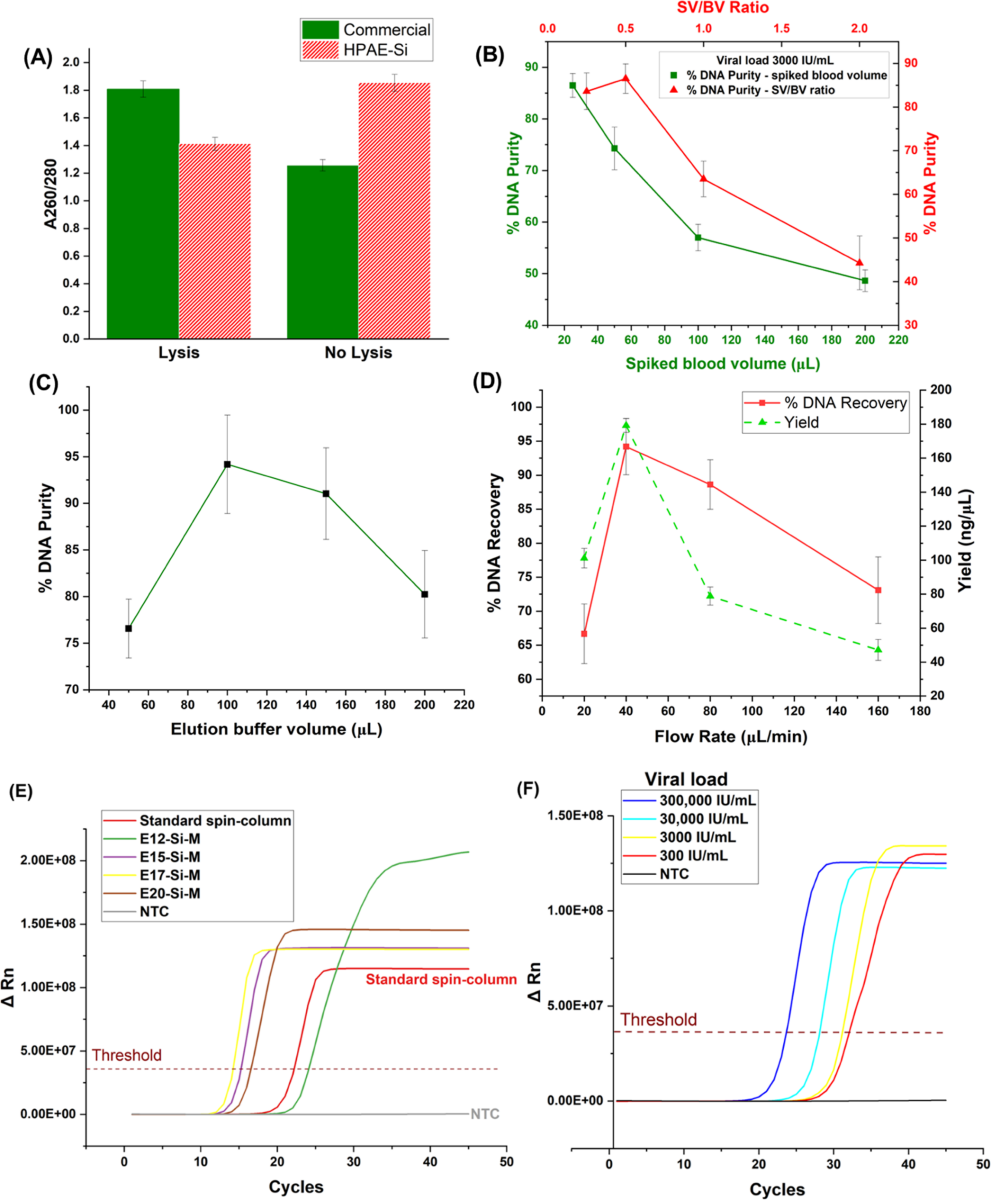
Recovery and purity of extracted DNA for (A) lysis conditions compared to Qiagen, (B) spiked blood and (C) elution buffer volumes, and (D) varying ratios of spiked blood and buffer ratio. (E) CMV FLOS-LAMP assay signal intensity curves of standard and PAE-modified membrane DNA extract. (F) performance of extracted DNA using the CMV FLOS-LAMP assay with the lowest limit of detection at 300 IU/mL viral load.

Commercial lysis reagents contain a detergent such as SDS or Triton X that can break open cells for isolating DNA for downstream processes in molecular biology. Sodium dodecyl sulfate (SDS) is inhibitory in most amplification processes and thus is not preferred for DNA extraction. Alternatively, Triton X is a nonionic detergent that can break the cell membrane without affecting amplification processes.^51^ PAE-modified membrane-based DNA extraction by directly treating 100 μL of blood with an equal volume of washing buffer had a higher absorbance ratio (1.8) when compared to pretreatment with a commercial lysis buffer (1.4, Figure 7A). Lysis buffers contain a high number of anionic detergents such as SDS, which can destabilize the charge around the HPAE-Si membrane, thus inhibiting the DNA binding process.^52^ Furthermore, as the primary role of lysis buffers is to release and denature cell membrane proteins, the presence of high amino acid content in the lysate reduced template DNA purity and showed no detectable signal when quantitative LAMP was performed.^53^ Thus, lysis and DNA binding were combined as a single wash step using a chaotropic salt solution with Triton X.

Blood sample volume and viral load can affect the amount of DNA available for binding and extraction due to the ratio of total DNA and contaminants (plasma components such as proteins). It was observed that for a fixed viral load of 3000 IU/mL, the purity of DNA recovered reduced with an increase in sample volume (Figure 7B). This could be due to the increased nonspecific binding of circulating DNA in plasma on the cationic surface while significant amounts of viral DNA were pumped out as waste. This was confirmed by collecting the filtrate waste and extracting DNA from it using a Qiagen Spin Kit, and the DNA yield was 132 ng/μL. A critical metric in the assay development of the HPAE-Si membrane extraction was the ratio of sample volume to wash buffer volume (SV/BV). As the area of the membrane is fixed and therefore the number of open cationic binding sites is limited, the ideal pH and salt concentration conditions driving DNA toward the binding sites are influenced by the volume of the buffer relative to the blood volume. This was apparent that as the SV/BV ratio increased, the buffer amount was insufficient to increase the positive charge density on the membrane surface and drive the exposed phosphate backbone of the DNA to bind. Thus, polymer cationic sites were occupied nonspecifically by contaminant biomolecules, resulting in a much lower DNA purity for the same HPAE-modified membrane, while an assay using an SV/BV ratio of 0.5 on the HPAE-modified membrane resulted in >90% DNA purity % (Figure 7B). The volume of elution buffer used for one cycle was found to be optimal at 100 μL and contaminants increased with increasing elution buffer volume (Figure 7C). However, in the case of viral loads <3000 IU/mL, increasing the number of elution cycles to 2 or 3 was found to increase DNA purity (from 77 to 92%), while the yield increased to 179 ng/μL; thus, the number of elution cycles depend on the expected viral load. Regarding the significance of heating, the following explanation is given. While the purity was found to be unaffected, the resulting DNA yield reduced from 176.2 ± 3.2 to 134.6 ± 4.4 ng/μL without the heating step. This is in line with previous research on silica-based extraction, as heating helps the membrane lyse more effectively and release cell contents onto the membrane.^54,55^

After recording the absorbance ratios at 260 and 280 nm and DNA yield, the extractant was used as template DNA for real-time FLOS-LAMP detection of CMV. 5 μL of template DNA was added to the LAMP mix (12.5 μL of Bst WarmStart master mix, 2.5 μL of primer mix, 5 μL of nuclease-free water). Compared to the positive control extracted using the Qiagen MiniElute Spin kit, the CMV positive signal from samples extracted on-chip using modified membranes (E12, E15, E17, E20) was above the fluorescence threshold and was detected (Figure 7E). Each sample was amplified in triplicates and the mean cycle time to detection was found to be 12 min (E15, E17, E20 modified) and 20 min (E12 modified). This cycle time variation could be attributed to the increased specificity of DNA binding, as E15, E17, and E20 modified membranes were able to improve the A260/A280 ratio to 1.7 ± 0.02 when compared to the E12 (1.52 ± 0.04) modified membrane. Performance evaluation of the LAMP assay using the extracted sample as a template showed a lower limit of detection of 300 IU/mL viral load (Figure 7F). All assays were repeated in triplicates and tested as five batches on different days for reproducibility (*p* < 0.05)**.

The on-chip modified silica membrane-based extraction and LAMP detection of CMV from blood was compared to commercial systems with integrated DNA extraction and amplification (Table 5). The purpose of this was to further the evidence of process simplification including time, instruments, sample preprocessing, and manual steps.

**Table 5:** Semiautomated Commercial Nucleic Acid Extraction Systems

name	sample type and volume	nucleic acid purification	manual steps	detection and turnaround time	refs
Cepheid GeneXpert MTB-RIF	sputum (after lysis 2 mL)	filter-based capture of bacilli and ultrasonic lysis	vortexing during lysis. Transfer of the sample into an Xpert MTB/RIF cartridge and loading the cartridge into the instrument	real-time reverse transcriptase PCR with fluorescent detection. 2 h	(27, 56, 57)
Abbott ID now SARS-CoV2	throat swab (200 μL Universal Transport Media)	no further purification	>1 cartridge transfers	real-time isothermal amplification with fluorescent detection. 13 min	(28, 58, 59)
Biofire (BioMerieux) FilmArray Blood Culture ID Panel	whole blood (200 μL)	binding on surface-functionalized magnetic beads	no manual steps. Each film array is for 1 sample, disposable, and does all three (extraction, PCR, detection) in the instrument	PCR detection with fluorescent probes. 1 h	(2, 60, 61)
NucliSENS EasyQ HIV-1 (BioMerieux) Automated Unit including NucliSENS easyMAG configuration NucliSENS EasyQ configuration Mini Strip Centrifuge	plasma (0.1 mL)	adsorption on magnetic silica beads	transfer of the sample from the bead-based extraction unit to the amplification unit	NASBA amplification with fluorescent probes. 3 h	(2, 62)
Alethia CMV (Meridian Biosciences)	saliva swab (1 mL Universal Transport Media)	no further purification	vortexing. Transfer of the processed sample to the instrument	LAMP with fluorescent detection. Under 1 h	(30)
AmpliVue C. difficile (Quidel)	stool (50 μL with lysis buffer)	no further purification	transfer of the lysed sample into the reaction tube. Placement of the reaction tube into the amplicon cartridge. Placement into the detection cassette	LAMP with fluorescent detection. Around 1.5 h	(29)
PAE-modified membrane	plasma (50 μL)	attachment on the membrane and elution	injection of the sample and buffers into the port. Transfer of the cartridge to the heating system	LAMP with fluorescent detection (integrated on chip/external). Extraction time—17–20 min. Total time to detection (including LAMP)—1.5 h	

## 4 Conclusions

A two-step DNA extraction microfluidic system was developed using HPAE-modified silica membranes and demonstrated for CMV detection from plasma. The synthesis and screening of HPAE coatings, modification of the membrane, and microfluidic chip fabrication methods are described, and the performance of the system was compared against commercially available silica spin column kits. GPTMS % and UV curing time were varied to identify the ideal conditions for all of the HPAEs used to coat the membranes to improve the coating process efficiency. The system coated with HPAE E15 (with a branched structure and the highest molecular weight) exhibited high DNA extraction performance. Using E15-modified membranes, an absorbance ratio (A260/280) of 1.89 and a DNA recovery of 94% with an average yield of 176.2 ± 3.2 ng/μL have been achieved from plasma without any pretreatment steps, thus providing a rapid and scalable nucleic acid extraction method that can be integrated with an on-chip amplification component. The fluorescent signal intensity detected from the LAMP assay using the extracted template with a minimal viral load of 300 IU/mL was comparable with conventionally extracted CMV DNA, with fewer manual steps and the need for no bulky instruments in a rapid manner (20 min). In this work, the application of HPAEs for nucleic acid capturing was demonstrated and provided an efficient method for low-resource on-chip extraction for commercial sample-to-result diagnostic devices. This work has also demonstrated the use of a proof-of-concept application of CMV detection as a highly sensitive, near-patient device for long-term viral load monitoring in transplant patients. Future work will include developing the system further to accommodate a wide variety of clinical specimens and automation to avoid the need for an external heating step.

## Supporting Information Available

Batch-to-batch variations in mean cycle time for detection of CMV from on-chip-extracted DNA using the HPAE-modified membrane against spin column-extracted DNA; batch-to-batch variations in DNA purity (absorbance ratio 260/280 nm) between the on-chip E15-modified membrane extractant and the standard spin column kit extractant; and absorbance data at 260 nm on a Nanodrop Lite using standard Acrometrix CMV dsDNA dilutions for calibration (PDF)

## Abbreviations

HCMV: human cytomegalovirus
PCR: polymerase chain reaction
LAMP: loop-mediated amplification
SPE: solid-phase extraction
RPM: revolutions per minute
PMMA: poly(methyl methacrylate)
PET: poly(ethylene terephthalate)
H/LPAE: hyperbranched/linear poly(β-amino ester)
MPA: morpholinopropylamine
PTTA: pentaerythritol tetraacrylate
GPTMS: (3-glycidyloxypropyl)trimethoxysilane
GPC: gel permeation chromatography
^1^H NMR: proton nuclear magnetic resonance
ATR-FTIR: attenuated total reflectance-Fourier transform infrared spectroscopy
SEM: scanning electron microscopy
EDTA: ethylenediaminetetraacetic acid

## References

[ref1] Hemmersbach-Miller M.; Alexander B. D.; Sudan D. L.; Pieper C.; Schmader K. E. Infections after kidney transplantation. Does age matter?. Clin. Transplant. 2019, 33, e13516 10.1111/ctr.13516. 30849194 PMC6465112

[ref2] Sachdeva S.; Davis R. W.; Saha A. K. Microfluidic Point-of-Care Testing: Commercial Landscape and Future Directions. Front. Bioeng. Biotechnol. 2021, 8, 1537 10.3389/fbioe.2020.602659. PMC7843572 33520958

[ref3] Jagannath A.; Cong H.; Hassan J.; Gonzalez G.; Gilchrist M. D.; Zhang N. Pathogen detection on microfluidic platforms: Recent advances, challenges, and prospects. Biosens. Bioelectron.: X 2022, 10, 100134 10.1016/j.biosx.2022.100134.

[ref4] Cong H.; Zhang N. Perspectives in translating microfluidic devices from laboratory prototyping into scale-up production. Biomicrofluidics 2022, 16, 021301 10.1063/5.0079045. 35350441 PMC8933055

[ref5] Pan W.; Wang X.; Ma X.; Chu Y. N.; Pang S.; Chen Y.; Guan X.; Zou B.; Wu Y.; Zhou G. Postsynthetic Modification of the Magnetic Zirconium–Organic Framework for Efficient and Rapid Solid-Phase Extraction of DNA. ACS Appl. Mater. Interfaces 2021, 13, 50309–50318. 10.1021/acsami.1c12622. 34652138

[ref6] Dhaliwal A. DNA extraction and purification. Mater. Methods 2013, 3, 191 10.13070/mm.en.3.191.

[ref7] Huang F.; Liang H. Adsorption behaviors of DNA/cation complexes on amino and silica chip surfaces: a dual polarization interferometry study. ACS Appl. Mater. Interfaces 2013, 5, 5025–5033. 10.1021/am400813x. 23697755

[ref8] Vandeventer P. E.; Lin J. S.; Zwang T. J.; Nadim A.; Johal M. S.; Niemz A. Multiphasic DNA adsorption to silica surfaces under varying buffer, pH, and ionic strength conditions. J. Phys. Chem. B 2012, 116, 5661–5670. 10.1021/jp3017776. 22537288 PMC3766398

[ref9] Chen S.; Chen X.; Du J.; Zhang Y.; Yang H. In In-Flow Extraction of RNA in Extracellular Vesicles Using a Silicon-Based Microfluidic Device, 2021 IEEE 34th International Conference on Micro Electro Mechanical Systems (MEMS); IEEE, 2021; pp 1015–1018.

[ref10] Petralia S.; Sciuto E. L.; Conoci S. A novel miniaturized biofilter based on silicon micropillars for nucleic acid extraction. Analyst 2017, 142, 140–146. 10.1039/C6AN02049F. 27917431

[ref11] Samla G.; Gan K. B.; Then S. M. Solid phase microextraction based micro-device for extraction of PCR amplifiable DNA. Exp. Theor. Nanotechnol. 2017, 1, 81–96. 10.56053/1.2.81.

[ref12] So H.; Lee K.; Murthy N.; Pisano A. P. All-in-one nanowire-decorated multifunctional membrane for rapid cell lysis and direct DNA isolation. ACS Appl. Mater. Interfaces 2014, 6, 20693–20699. 10.1021/am506153y. 25420232 PMC4264858

[ref13] Park B. H.; Oh S. J.; Jung J. H.; Choi G.; Seo J. H.; Kim D. H.; Lee E. Y.; Seo T. S. An integrated rotary microfluidic system with DNA extraction, loop-mediated isothermal amplification, and lateral flow strip based detection for point-of-care pathogen diagnostics. Biosens. Bioelectron. 2017, 91, 334–340. 10.1016/j.bios.2016.11.063. 28043075

[ref14] Günal G.; Kip Ç.; Öğüt S. E.; Usta D. D.; Şenlik E.; Kibar G.; Tuncel A. Human genomic DNA isolation from whole blood using a simple microfluidic system with silica-and polymer-based stationary phases. Mater. Sci. Eng.: C 2017, 74, 10–20. 10.1016/j.msec.2016.12.118. 28254272

[ref15] Zhang J.; Su X.; Xu J.; Wang J.; Zeng J.; Li C.; Chen W.; Li T.; Min X.; Zhang D.; et al. A point of care platform based on microfluidic chip for nucleic acid extraction in less than 1 minute. Biomicrofluidics 2019, 13, 034102 10.1063/1.5088552. 31123534 PMC6506337

[ref16] Choi Y.; Song Y.; Kim Y. T.; Kim H.; Park Y. M.; Lee S. J.; Im S. G.; Lee K. G. All-in-one pumpless portable genetic analysis microsystem for rapid naked-eye detection. Sens. Actuators, B 2021, 344, 130307 10.1016/j.snb.2021.130307.

[ref17] Liu H.-W.; Hu Y.; Ren Y.; Nam H.; Santos J. L.; Ng S.; Gong L.; Brummet M.; Carrington C. A.; Ullman C. G.; et al. Scalable purification of plasmid DNA nanoparticles by tangential flow filtration for systemic delivery. ACS Appl. Mater. Interfaces 2021, 13, 30326–30336. 10.1021/acsami.1c05750. 34162211 PMC9701136

[ref18] Mahalanabis M.; Al-Muayad H.; Kulinski M. D.; Altman D.; Klapperich C. M. Cell lysis and DNA extraction of gram-positive and gram-negative bacteria from whole blood in a disposable microfluidic chip. Lab Chip 2009, 9, 2811–2817. 10.1039/b905065p. 19967118

[ref19] Liu Q.; Nam J.; Kim S.; Lim C. T.; Park M. K.; Shin Y. Two-stage sample-to-answer system based on nucleic acid amplification approach for detection of malaria parasites. Biosens. Bioelectron. 2016, 82, 1–8. 10.1016/j.bios.2016.03.050. 27031184

[ref20] Shin Y.; Lim S. Y.; Lee T. Y.; Park M. K. Dimethyl adipimidate/Thin film Sample processing (DTS); A simple, low-cost and versatile nucleic acid extraction assay for downstream analysis. Sci. Rep. 2015, 5, 14127 10.1038/srep14127. 26370251 PMC4569962

[ref21] Jin C. E.; Koo B.; Lee E. Y.; Kim J. Y.; Kim S.-H.; Shin Y. Simple and label-free pathogen enrichment via homobifunctional imidoesters using a microfluidic (SLIM) system for ultrasensitive pathogen detection in various clinical specimens. Biosens. Bioelectron. 2018, 111, 66–73. 10.1016/j.bios.2018.04.001. 29653418 PMC7125596

[ref22] Paul R.; Ostermann E.; Wei Q. Advances in point-of-care nucleic acid extraction technologies for rapid diagnosis of human and plant diseases. Biosens. Bioelectron. 2020, 169, 112592 10.1016/j.bios.2020.112592. 32942143 PMC7476893

[ref23] Chang C.-M.; Chang W.-H.; Wang C.-H.; Wang J.-H.; Mai J. D.; Lee G.-B. Nucleic acid amplification using microfluidic systems. Lab Chip 2013, 13, 1225–1242. 10.1039/c3lc41097h. 23407669

[ref24] Emaus M. N.; Varona M.; Eitzmann D. R.; Hsieh S.-A.; Zeger V. R.; Anderson J. L. Nucleic acid extraction: fundamentals of sample preparation methodologies, current advancements, and future endeavors. TrAC, Trends Anal. Chem. 2020, 130, 115985 10.1016/j.trac.2020.115985.

[ref25] Marshall L. A.; Wu L. L.; Babikian S.; Bachman M.; Santiago J. G. Integrated printed circuit board device for cell lysis and nucleic acid extraction. Anal. Chem. 2012, 84, 9640–9645. 10.1021/ac302622v. 23046297

[ref26] Kim J.; Johnson M.; Hill P.; Gale B. K. Microfluidic sample preparation: cell lysis and nucleic acid purification. Integr. Biol. 2009, 1, 574–586. 10.1039/b905844c. 20023774

[ref27] Dorman S. E.; Chihota V. N.; Lewis J. J.; Shah M.; Clark D.; Grant A. D.; Churchyard G. J.; Fielding K. L. Performance characteristics of the Cepheid Xpert MTB/RIF test in a tuberculosis prevalence survey. PLoS ONE 2012, 7, e43307 10.1371/journal.pone.0043307. 22905254 PMC3419700

[ref28] Vashist S. K. In vitro diagnostic assays for COVID-19: recent advances and emerging trends. Diagnostics 2020, 10, 202 10.3390/diagnostics10040202. 32260471 PMC7235801

[ref29] Deak E.; Miller S.; Humphries R. Comparison of Illumigene, Simplexa, and AmpliVue Clostridium difficile molecular assays for diagnosis of C. difficile infection. J. Clin. Microbiol. 2014, 52, 960–963. 10.1128/JCM.02354-13. 24352999 PMC3957777

[ref30] Gantt S.; Goldfarb D. M.; Park A.; Rawlinson W.; Boppana S. B.; Lazzarotto T.; Mertz L. M. Performance of the Alethia CMV assay for detection of cytomegalovirus by use of neonatal saliva swabs. J. Clin. Microbiol. 2020, 58, e01951-19 10.1128/JCM.01951-19. 31969426 PMC7098765

[ref31] Liu Y.; Li Y.; Keskin D.; Shi L. Poly (β-Amino Esters): synthesis, formulations, and their biomedical applications. Adv. Healthcare Mater. 2019, 8, 1801359 10.1002/adhm.201800995. 30549448

[ref32] Lynn D. M.; Langer R. Degradable poly (β-amino esters): synthesis, characterization, and self-assembly with plasmid DNA. J. Am. Chem. Soc. 2000, 122, 10761–10768. 10.1021/ja0015388.

[ref33] Akinc A.; Lynn D. M.; Anderson D. G.; Langer R. Parallel synthesis and biophysical characterization of a degradable polymer library for gene delivery. J. Am. Chem. Soc. 2003, 125, 5316–5323. 10.1021/ja034429c. 12720443

[ref34] Anderson D. G.; Tweedie C. A.; Hossain N.; Navarro S. M.; Brey D. M.; Van Vliet K. J.; Langer R.; Burdick J. A. A combinatorial library of photocrosslinkable and degradable materials. Adv. Mater. 2006, 18, 2614–2618. 10.1002/adma.200600529.

[ref35] Zhou D.; Cutlar L.; Gao Y.; Wang W.; O’Keeffe-Ahern J.; McMahon S.; Duarte B.; Larcher F.; Rodriguez B. J.; Greiser U. The transition from linear to highly branched poly (β-amino ester) s: Branching matters for gene delivery. Sci. Adv. 2016, 2, e1600102 10.1126/sciadv.1600102. 27386572 PMC4928911

[ref36] Zhang H.; Xu Y.; Fohlerova Z.; Chang H.; Iliescu C.; Neuzil P. LAMP-on-a-chip: Revising microfluidic platforms for loop-mediated DNA amplification. TrAC, Trends Anal. Chem. 2019, 113, 44–53. 10.1016/j.trac.2019.01.015. PMC7112807 32287531

[ref37] Gadkar V. J.; Goldfarb D. M.; Gantt S.; Tilley P. A. G. Real-time detection and monitoring of loop mediated amplification (LAMP) reaction using self-quenching and de-quenching fluorogenic probes. Sci. Rep. 2018, 8, 5548 10.1038/s41598-018-23930-1. 29615801 PMC5883045

[ref38] Wang X.; Yan C.; Wang X.; Zhao X.; Shi C.; Ma C. Integrated silica membrane–based nucleic acid purification, amplification, and visualization platform for low-cost, rapid detection of foodborne pathogens. Anal. Bioanal. Chem. 2020, 412, 6927–6938. 10.1007/s00216-020-02823-1. 32712814

[ref39] Shirosaki Y.; Tsuru K.; Hayakawa S.; Osaka A.; Lopes M. A.; Santos J. D.; Fernandes M. H. In vitro cytocompatibility of MG63 cells on chitosan-organosiloxane hybrid membranes. Biomaterials 2005, 26, 485–493. 10.1016/j.biomaterials.2004.02.056. 15276356

[ref40] Zhang H.; Liu X.; Li T.; Han X. Miscible organic solvents soak bonding method use in a PMMA multilayer microfluidic device. Micromachines 2014, 5, 1416–1428. 10.3390/mi5041416.

[ref41] Hall Sedlak R.; Castor J.; Butler-Wu S. M.; Chan E.; Cook L.; Limaye A. P.; Jerome K. R. Rapid detection of human cytomegalovirus UL97 and UL54 mutations directly from patient samples. J. Clin. Microbiol. 2013, 51, 2354–2359. 10.1128/JCM.00611-13. 23678068 PMC3697653

[ref42] Dioverti M. V.; Lahr B. D.; Germer J. J.; Yao J. D.; Gartner M. L.; Razonable R. R. Comparison of standardized cytomegalovirus (CMV) viral load thresholds in whole blood and plasma of solid organ and hematopoietic stem cell transplant recipients with CMV infection and disease. Open Forum Infect. Dis. 2017, 4, ofx143 10.1093/ofid/ofx143. 28852681 PMC5570102

[ref43] Health C. A. f. D. a. T. i. Clinical Review Report: Letermovir (Prevymis): (Merck Canada Inc.): Indication: For the prophylaxis of cytomegalovirus (CMV) infection in adult CMV-seropositive recipients (R+) of an allogeneic hematopoietic stem cell transplant [Internet]. 2018. 30942986

[ref44] Razonable R. R.; Hayden R. T. Clinical utility of viral load in management of cytomegalovirus infection after solid organ transplantation. Clin. Microbiol. Rev. 2013, 26, 703–727. 10.1128/CMR.00015-13. 24092851 PMC3811235

[ref45] Boom R.; Sol C.; Salimans M.; Jansen C.; Wertheim-van Dillen P.; van der Noordaa J. Rapid and simple method for purification of nucleic acids. J. Clin. Microbiol. 1990, 28, 495–503. 10.1128/jcm.28.3.495-503.1990. 1691208 PMC269651

[ref46] Marko M. A.; Chipperfield R.; Birnboim H. C. A procedure for the large-scale isolation of highly purified plasmid DNA using alkaline extraction and binding to glass powder. Anal. Biochem. 1982, 121, 382–387. 10.1016/0003-2697(82)90497-3. 6179438

[ref47] Myakishev M. V.; Kapanadze G. I.; Shaikhayev G. O.; Georgiev G. P.; Beritashvili D. R. Extraction of DNA from the Whole Blood by Silica Gel; Institute of Gene Biology: Moscow, 1995.

[ref48] Kamra T.; Chaudhary S.; Xu C.; Johansson N.; Montelius L.; Schnadt J.; Ye L. Covalent immobilization of molecularly imprinted polymer nanoparticles using an epoxy silane. J. Colloid Interface Sci. 2015, 445, 277–284. 10.1016/j.jcis.2014.12.086. 25626133

[ref49] Mousavi M.; Fini E. Silanization Mechanism of Silica Nanoparticles in Bitumen Using 3-Aminopropyl Triethoxysilane (APTES) and 3-Glycidyloxypropyl Trimethoxysilane (GPTMS). ACS Sustainable Chem. Eng. 2020, 8, 3231–3240. 10.1021/acssuschemeng.9b06741.

[ref50] Al balawi A. N.; Yusof N. A.; Kamaruzaman S.; Mohammad F.; Wasoh H.; Al Abbosh K. F.; Al-Lohedan H. A. Synthesis, Characterization, and Application of Poly(4,4′-Cyclohexylidene Bisphenol Oxalate) for Solid-Phase Extraction of DNA. BioMed. Res. Int. 2019, 2019, 7064073 10.1155/2019/7064073. 30868072 PMC6379882

[ref51] Schrader C.; Schielke A.; Ellerbroek L.; Johne R. PCR inhibitors–occurrence, properties and removal. J. Appl. Microbiol. 2012, 113, 1014–1026. 10.1111/j.1365-2672.2012.05384.x. 22747964

[ref52] Hill C.; Abdullahi W.; Crossman M.; Griffiths P. C. Using polymer–surfactant charge ratio to control synergistic flocculation of anionic particulate dispersions. Polymers 2022, 14, 3504 10.3390/polym14173504. 36080579 PMC9460132

[ref53] Rossen L.; Nørskov P.; Holmstrøm K.; Rasmussen O. F. Inhibition of PCR by components of food samples, microbial diagnostic assays and DNA-extraction solutions. Int. J. Food Microbiol. 1992, 17, 37–45. 10.1016/0168-1605(92)90017-W. 1476866

[ref54] Vandeventer P. E.; Mejia J.; Nadim A.; Johal M. S.; Niemz A. DNA adsorption to and elution from silica surfaces: influence of amino acid buffers. J. Phys. Chem. B 2013, 117, 10742–10749. 10.1021/jp405753m. 23931415 PMC4097040

[ref55] BioLabs N. E. https://international.neb.com/faqs/2018/11/02/how-important-is-it-to-pre-heat-the-elution-buffer-to-60c-would-56c-be-ok. (accessed 9 May 2023).

[ref56] Shinnick T. M.; Starks A. M.; Alexander H. L.; Castro K. G. Evaluation of the Cepheid Xpert MTB/RIF assay. Expert Rev. Mol. Diagn. 2015, 15, 9–22. 10.1586/14737159.2015.976556. 25373876 PMC5875728

[ref57] Lawn S. D.; Nicol M. P. Xpert MTB/RIF assay: development, evaluation and implementation of a new rapid molecular diagnostic for tuberculosis and rifampicin resistance. Future Microbiol. 2011, 6, 1067–1082. 10.2217/fmb.11.84. 21958145 PMC3252681

[ref58] Cradic K.; Lockhart M.; Ozbolt P.; Fatica L.; Landon L.; Lieber M.; Yang D.; Swickard J.; Wongchaowart N.; Fuhrman S.; Antonara S. Clinical evaluation and utilization of multiple molecular in vitro diagnostic assays for the detection of SARS-CoV-2. Am. J. Clin. Pathol. 2020, 154, 201–207. 10.1093/ajcp/aqaa097. 32462195 PMC7314271

[ref59] Mitchell S. L.; George K. S. Evaluation of the COVID19 ID NOW EUA assay. J. Clin. Virol. 2020, 128, 104429 10.1016/j.jcv.2020.104429. 32425657 PMC7227587

[ref60] Oeschger T.; McCloskey D.; Kopparthy V.; Singh A.; Erickson D. Point of care technologies for sepsis diagnosis and treatment. Lab Chip 2019, 19, 728–737. 10.1039/C8LC01102H. 30724931 PMC6392004

[ref61] FilmArray B. https://www.youtube.com/watch?v=KjAeOzTL1wo. (accessed 8 March 2023).

[ref62] Tanriverdi S.; Chen L.; Chen S. A rapid and automated sample-to-result HIV load test for near-patient application. J. Infect. Dis. 2010, 201, S52–S58. 10.1086/650387. 20225947

