# Supplementary material for: UV-Assisted Hyperbranched Poly(β-amino ester) Modification of a Silica Membrane for Two-Step Microfluidic DNA Extraction from Blood
Source: ACS Appl Mater Interfaces. 2023 Jun 15;15(26):31159–72. doi: 10.1021/acsami.3c03523 (PMC10326802; doi:10.1021/acsami.3c03523)
Supplement: Supplementary file 1 — am3c03523_si_001.pdf [file am3c03523_si_001.pdf]

Supporting Information

UV-assisted hyperbranched poly(β-amino ester) modification of silica membrane for 2-step microfluidic DNA extraction from blood

Akshaya Jagannath, Yinghao Li, Hengji Cong, Jaythoon Hassan, Gabriel Gonzalez, Wenxin Wang, Nan Zhang, Michael D. Gilchrist

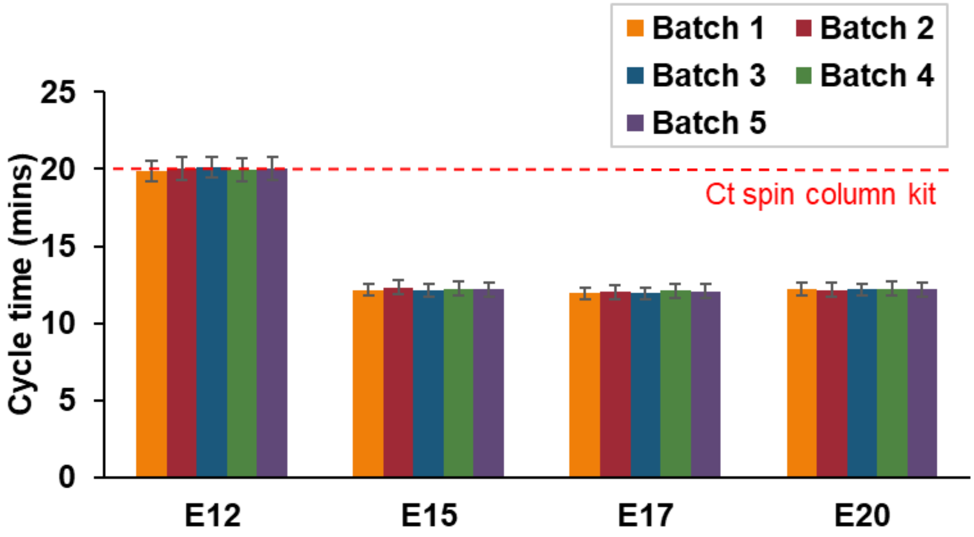

Figure S1: Batch-to-batch variations in mean cycle time for detection of CMV from on-chip extracted DNA using HPAE-modified-membrane against spin column extracted DNA

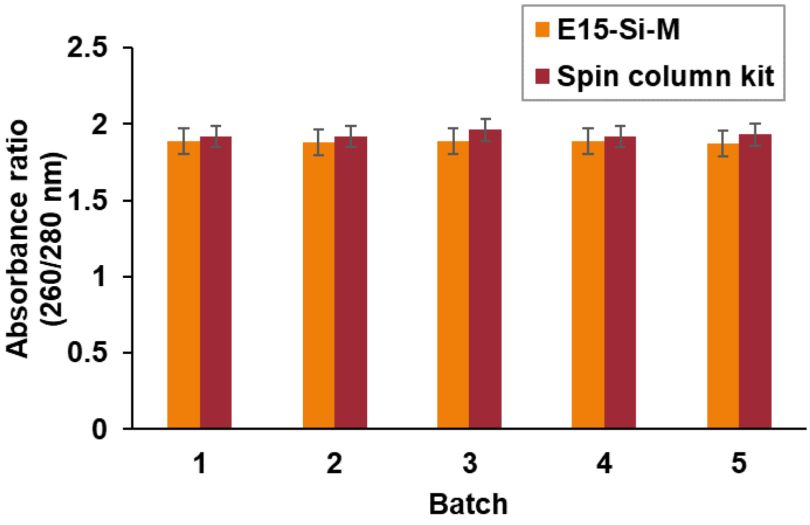

Figure S2: Batch-to-batch variations in DNA purity (absorbance ratio 260/280 nm) between on-chip E15-modified membrane extractant and standard spin column kit extractant

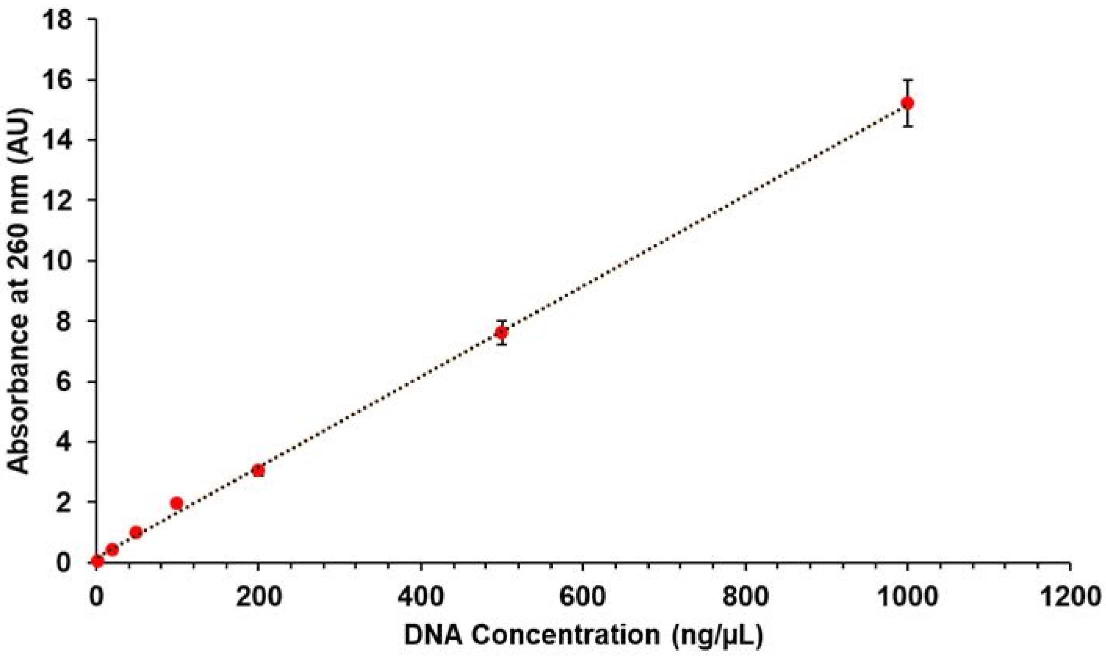

Figure S3: Absorbance at 260 nm on Nanodrop Lite using standard Acrometrix™ CMV dsDNA dilutions for calibration.
